# Inhaled nanomaterials and the respiratory microbiome: clinical, immunological and toxicological perspectives

**DOI:** 10.1186/s12989-018-0282-0

**Published:** 2018-11-20

**Authors:** Tuang Yeow Poh, Nur A’tikah Binte Mohamed Ali, Micheál Mac Aogáin, Mustafa Hussain Kathawala, Magdiel Inggrid Setyawati, Kee Woei Ng, Sanjay Haresh Chotirmall

**Affiliations:** 1 Translational Respiratory Research Laboratory, Lee Kong Chian School of Medicine, Nanyang Technological University, Level 12, Clinical Sciences Building, 11 Mandalay Road, Singapore, 308232 Singapore; 2 School of Materials Science and Engineering, Nanyang Technological University, Block N4.1, 50 Nanyang Avenue, Singapore, 639798 Singapore

**Keywords:** Nanoparticle, Nanotoxicology, Nanomaterial, Respiratory disease, Microbiome

## Abstract

Our development and usage of engineered nanomaterials has grown exponentially despite concerns about their unfavourable cardiorespiratory consequence, one that parallels ambient ultrafine particle exposure from vehicle emissions. Most research in the field has so far focused on airway inflammation in response to nanoparticle inhalation, however, little is known about nanoparticle-microbiome interaction in the human airway and the environment. Emerging evidence illustrates that the airway, even in its healthy state, is not sterile. The resident human airway microbiome is further altered in chronic inflammatory respiratory disease however little is known about the impact of nanoparticle inhalation on this airway microbiome. The composition of the airway microbiome, which is involved in the development and progression of respiratory disease is dynamic, adding further complexity to understanding microbiota-host interaction in the lung, particularly in the context of nanoparticle exposure. This article reviews the size-dependent properties of nanomaterials, their body deposition after inhalation and factors that influence their fate. We evaluate what is currently known about nanoparticle-microbiome interactions in the human airway and summarise the known clinical, immunological and toxicological consequences of this relationship. While associations between inhaled ambient ultrafine particles and host immune-inflammatory response are known, the airway and environmental microbiomes likely act as intermediaries and facilitate individual susceptibility to inhaled nanoparticles and toxicants. Characterising the precise interaction between the environment and airway microbiomes, inhaled nanoparticles and the host immune system is therefore critical and will provide insight into mechanisms promoting nanoparticle induced airway damage.

## Background

A growing proclivity for nanomaterials coupled to their increased emission as by-products of novel technologies and industrial processes has led to concerns over their potential toxic effect in humans and strategies to circumvent it [1, 2]. Exposure to nanomaterials through inhalation has received particular attention [3, 4]. The surfaces of large (conducting) airways are lined by ciliated bronchial epithelial cells and mucus producing goblet cells. In bronchioles, epithelial and Clara cells predominate. All epithelial cells reside on a basement membrane. The air-blood barrier at the alveolus consists of type I epithelium and surfactant-producing type II cells. This barrier, measuring 0.1–0.2 μm, is the most permeable barrier in the human body. Nanoparticles that are small enough, can reach the lower airways and gain access to the air-blood barrier, while larger particles (> 5 μm) remain trapped in the upper airways, where the epithelial lining is thicker and cells are blanketed by protective mucus. The high surface area, rapid absorption due to vascularization and circumvention of the first pass effect allows nanoparticles to freely cross the air-blood barrier [5]. Epidemiological human studies show that exposure to ultrafine particles (< 2.5 μm) in the air increases pulmonary morbidity and mortality [6–10]. Further, pulmonary fibrosis and pleural granuloma formation are reported in workers weeks after exposure to polyacrylate nanoparticles where particles are detectable in the cytoplasm and nucleus of pneumocytes and mesothelial cells [11]. Animal studies further demonstrate that NPs at equivalent mass doses cause inflammation and cross the alveolar barrier in higher numbers compared to larger particles [12]. Concurrently, advances in culture-independent DNA sequencing of the human microbiome has shed light on the importance of microbe-host interactions which are reshaping our understanding of human disease and toxicology [13–17]. While clear advances have been made in both fields, our understanding of the interaction between inhaled nanomaterials and the lung microbiome is lacking and many unanswered questions persist [18]. Here we aim to address this knowledge gap by assessing the current state of the literature with regard to microbe-nanomaterial interaction and the potential clinical implications for toxicology and risk of respiratory disease.

### Literature search strategy

We undertook a literature review by searching PubMed up to October 1st 2018 for relevant articles using the following search string; “(nanoparticles OR nanotoxicology OR nanomaterials) AND (bacteria OR virus OR fungi OR microbiome) AND (lung OR respiratory OR pulmonary).” This returned 755 entries since 2002 including 686 original research articles. We assessed these articles and included only those with direct relevance to respiratory disease and the microbiome via toxicological effects related to inhalation of nanomaterials, which formed the basis of this review.

#### Introduction: Nanomaterials and toxicology

**Fig. 1:**
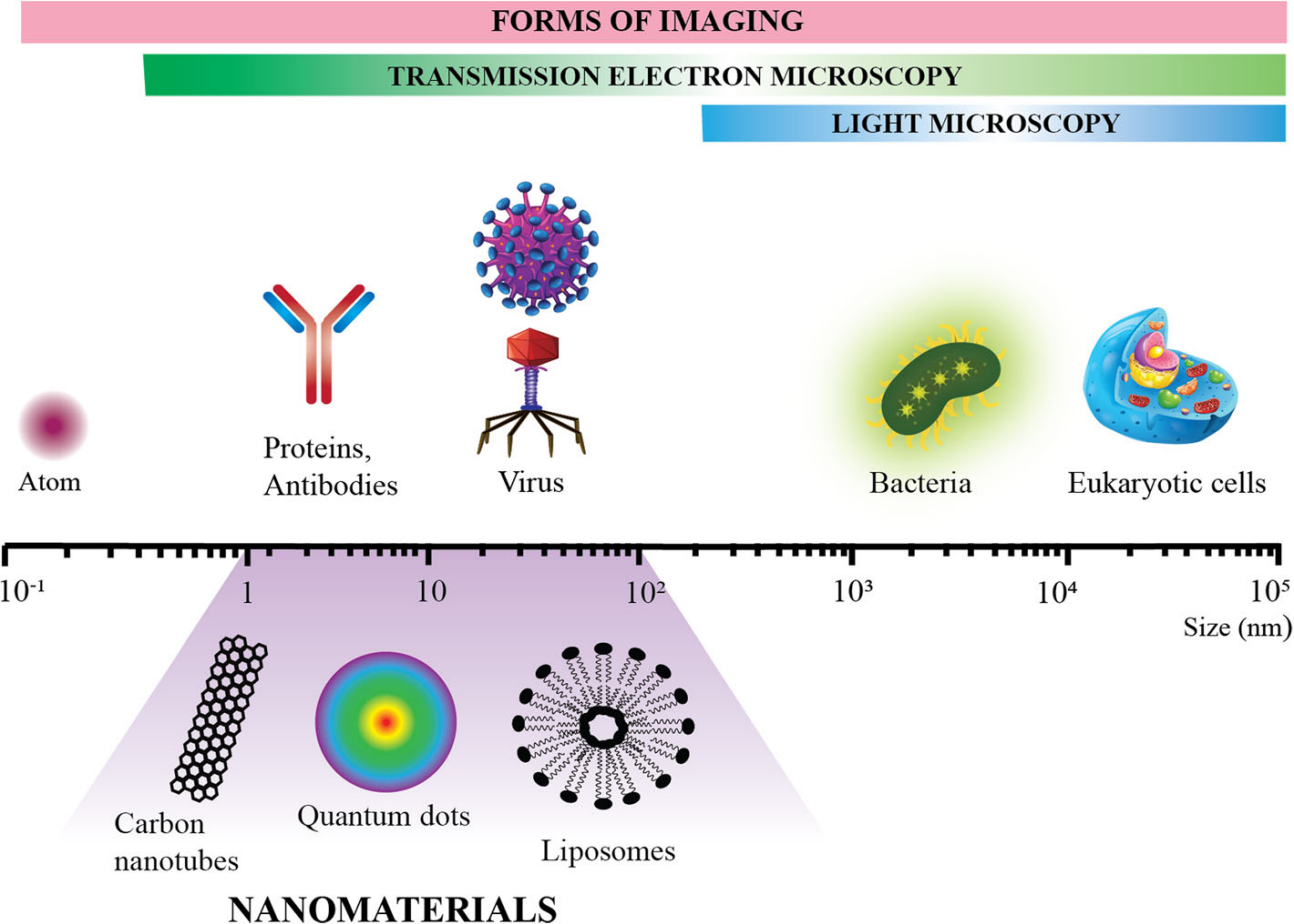
Relative size comparison of nanomaterials, microbiological and other biological entities. Bodies visible by light and transmission electron microscopy are indicated and a scale bar denotes the size range of the respective biological entities and nanomaterials (1-100 nm)

**Fig. 2:**
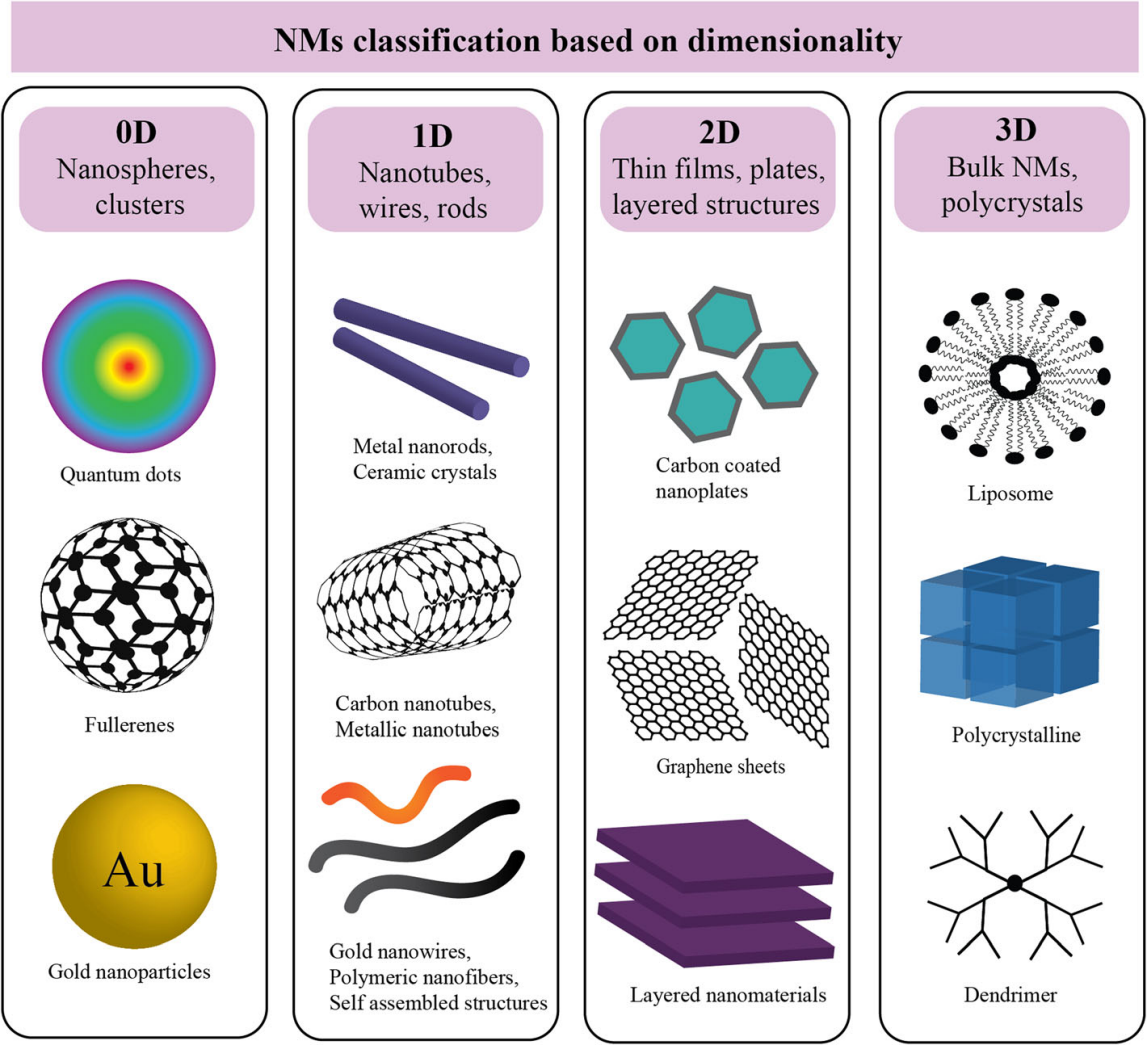
Schematic illustrating the relative dimensions of nanoparticles with examples of each category. Nanomaterials (NMs) exist in different dimensionality and current classification schemes of nano-structured materials (NSM) are proposed as zero (OD), one (1D), two (2D) and three dimensional (3D). 3D nanocomposites form when two or more materials with individual properties act synergistically to create a composite with unique properties

Nanoscience and nanotechnology is defined by the Royal Society and Academy of Engineering as “the study of phenomena and manipulation of materials at atomic, molecular and macromolecular scales, where the properties differ significantly from those at a larger scale” [1, 19]. Despite this definition, the term ‘nano’ is often inconsistently used (even in scientific discourse) and in this review, only materials with at least one dimension measuring less than 100 nm (nm) are considered nanomaterials (NMs) (Fig. 1) [20]. In the broadest sense, NMs are divided based on their number of dimensions in nanoscale (Fig. 2) [21]. Within each classification, NMs are further characterised based on organic or inorganic composition, the former being NMs synthesized from polymers, phospholipids, proteins and their hybrids, and the latter metals, metal oxides, carbon-based, semiconductors and quantum dots.

The success of NMs is a consequence of their high surface area to volume/mass ratio supporting greater surface reactivity. This overcomes material-limiting processes in applications such as catalysis, structural strengthening and biomedicine [22–27]. In addition, their small size permits access to the most inaccessible spaces which confers advantages for applications requiring penetration of physical barriers or the delivery of agents [28–33]. Importantly, these very properties making widespread use attractive are also implicated in explaining their harmful and toxicological consequence for humans and environmental health. The ability of NMs to penetrate biological barriers including the lung epithelium or the blood-brain barrier for instance, permits elicitation of a biological response, one differing between body compartments and conferring potentially toxic outcomes. Scientific research focused on nanomaterial-induced toxicity has struggled to keep pace with the rapid advances in nanotechnology. Nonetheless, an improved awareness and greater research investment in the field over the last two decades have made terms such as ‘nanotoxicology’ and ‘nanosafety’ commonplace in the scientific literature. While the direct impact of NM exposure on human health is being intensely studied, especially in terms of direct interaction with human cells and tissues; to ensure a comprehensive understanding of their impact on human health, we must consider the consequences of indirect exposure, particularly secondary interactions with microbiota, resident in the lungs (the microbiome) and that in the surrounding environment.

#### The host and environmental microbiome

Over the last decade, novel facets of human physiology have been ascribed to the microbiome: the collection of genetic material of all microbes living on and inside the human body. Our existence therefore as ‘holobiont’ individuals composed of human and microbial cells is now apparent as key homeostatic functions of the microbiome such as nutrient production, polysaccharide digestion, pathogen evasion, immune regulation and detoxification are all critical to the maintenance of health and where dysfunctional, result in disease [14–17, 34–37]. While much remains to be learned about the human microbiome, our current understanding acknowledges its essential role in biological functioning including toxicology [13]. Insight gained from microbiome analysis challenges our accepted and simplistic definitions of toxicity, shifting from one focused solely on human cells, to that of toxicity toward ‘protective microbes’ that are important in maintaining a united ecosystem. Triclosan for instance, a once recognised breakthrough antimicrobial is now viewed with apprehension due to its potential for selecting out multidrug resistant pathogens and for its negative effects on the infant gut microbiome [38, 39]. Similarly, concerns have been raised about the effects of inhaled NPs on the lung microbiome given their ability for aerosolisation and to penetrate the lung’s epithelium depending upon its physicochemical characteristics [3, 4]. The study of the lung microbiome however significantly lags behind that of the gut, predominantly due to its exclusion from the human microbiome project [40]. Long considered to be sterile, the lung has suffered from a lack of dedicated microbiome studies and its microbial constituents have only recently been described by our group and others [40]. The lung has a thriving ecosystem of microbial residents including an emerging core set of taxa that comprise the healthy lung microbiome [41]. Rather than sterile sites that become infected by invading microbes, a new model of lung disease is emerging that suggests a careful balancing and selection of a group of core microbial constituents in the lung, which, if perturbed, leads to pathological signatures and disease via a process known as ‘dysbiosis’ [40]. In such circumstances the balanced and diverse ecosystem of the lung becomes unstable and overrun with particular pathogenic members that shift the balance toward less favourable disease states, the triggers of which are now the subject of intense research efforts. This includes studying gut-lung crosstalk driven by microbiome composition at both sites [42]. The healthy airway appears to exhibit a core microbiota of several bacterial genera including *Prevotella, Streptococcus* and *Veillonella* as well as *Corynebacterium Haemophilus*, *Neisseria* and *Actinobacteria* that vary dependent on the population and specific airway site under investigation [16, 40]. Significant shifts in these microbial consortia towards domination by genera including pathogenic members such as *Pseudomonas*, *Staphylococcus*, *Haemophilus* and *Moraxella* are noted in chronic respiratory disease states including chronic obstructive pulmonary disease (COPD), asthma, idiopathic pulmonary fibrosis (IPF) and cystic fibrosis (CF) [41]. Major chronic respiratory diseases have now been the subject of many culture-independent microbiome studies revealing varying degrees of dysbiosis associated with clinical outcome or host immune response [43–45]. In this context, assessing the impact of inhaled NPs on lung microbiome architecture is pivotal to developing accurate, representative, holistic and cogent models of NP toxicity [40]. With greater application of NPs and NMs in everyday life, their unquestionable benefits must be carefully balanced against potential deleterious effects on the host (both human and microbial cells) in order to advance more robust paradigms for the nanotoxicology field.

In addition to the host microbiome, the microbiome of the built environment (the environmental microbiome) must also be considered for potential interaction with ambient NPs. Characterising the environmental microbiome is a burgeoning area of scientific research, with major implications for structural engineering and human health and disease. Ecological (and therefore microbiome) change begins once new structures are constructed and indoor surfaces and systems decontaminated [46–48]. Materials and compounds used for construction and decontamination therefore shape the microbial constituents of the built environment where humans live and work [49–51]. Advances in high-throughput sequencing technologies now allow culture-independent insight into the microbial constituents of the indoor and outdoor environment revealing the vast diversity of microorganisms and an understanding of microbial ecology within an increasingly ‘engineered’ and urbanised world [52, 53].

Microbes can persist on surfaces, in air, and within water systems. Their presence and viability depend on the characteristics and dynamic interactions of the built environment, the microbial community and the human occupants within it. Different indoor environments have distinct microbiome signatures, a characteristic extending to include different rooms within the same building [54–59]. Microbes carried on living organisms inhabiting the environment further contribute to the surrounding environmental microbiome. This is evidenced by human occupancy shaping the overall microbial community structure, particularly in heavily occupied or poorly ventilated areas. Indoor air and surfaces are dominated by major human-associated microbes including *Actinobacteria, Bacteroidetes, Firmicutes, and Proteobacteria* [51, 60, 61]. Microbial load and diversity within a fixed indoor environment may be further influenced by other factors including the outdoor environment; ventilation; air-conditioning and plumbing systems; and the presence of mould and dust [50]. Therefore, while host microbiomes contribute to the environment, the environmental microbiome similarly influences the host in beneficial or disadvantageous ways shaping human health or promoting disease [51, 57]. Dust-associated microbes such as *Firmicutes* and *Bacteroidetes* may predispose to asthma and allergy while dust exposure alone can have beneficial effects for the gut microbiome and host immunity [62, 63]. Water-derived opportunistic pathogens including *Pseudomonas aeruginosa, Legionella pneumophila* and *Mycobacterium avium* grow as biofilms in plumbing systems, and exposure causes a wide array of skin and pulmonary infections [64–68]. Interestingly, an association between the microbial composition of drinking water and the gut microbiome is demonstrated in germ-free mouse models further affirming the influence of the environmental microbiome on human health [69].

NMs are being increasingly used in many fields relevant to the built environment. In this light, while such materials offer benefits including reduced vibration through self-compacting concretes and improved energy efficiency from thermally insulated windows, there is a general lack of appreciation of the influence that such NMs have upon the built environment and the occupant’s microbiota with potential public health consequences [49, 70]. Use of nanosilver coating for its anti-microbial properties for instance risks selecting out drug-resistant microbes and engineered NMs such as nanotitanium dioxide from consumer products, paints and clothing can pass through the water treatment process and are found in tap water at significant and potentially harmful concentrations [71, 72]. The continued growth of NM use highlights the urgent need to better understand their risks, especially as direct human exposure to NMs occurs in everyday life. Dedicated work focused on the characteristics and dynamic interaction between NMs of the built environment and associated environmental microbiota are required as they likely to influence the human (lung) microbiome and have consequences for maintaining health and promoting disease. Further work into intrinsically safer NM design, through structural and/or chemical alteration, should be undertaken to further reduce potential health risks.

#### Nanomaterial-Microbiome Interactions

To date, mechanistic insight into the interaction between NPs and microbes is limited [73]. Current approaches for understanding NM-microbe interactions are generally indirect; most studies monitor changes in microbial activity or survival in response to NMs exposure, outcome measures that may not represent the complete picture [74, 75]. Additionally, reports documenting these interactions remain limited and largely focused on antimicrobial nanomedicine, a field cataloguing such interactions in detail [76–78].

**Fig. 3:**
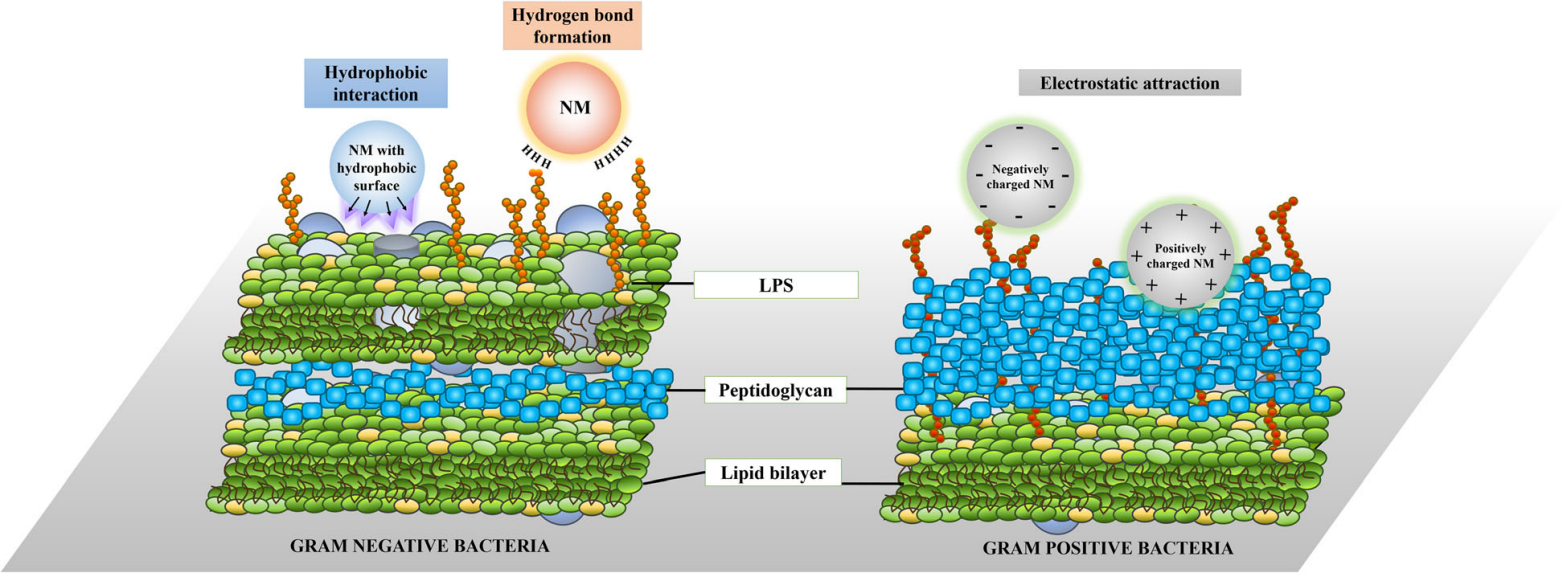
Summary of possible mechanisms of interaction between nanoparticles and the cell surface of gram-negative (left panel) and gram-positive (right panel) bacteria. Potential interaction between nanomaterials and microbial cells are illustrated. Hydrophobic interaction and hydrogen bond formation are predominant forces promoting the attachment of nanomaterials to the cell membrane of gram-negative bacteria. This is driven by the interaction between lipopolysaccharides (LPS) and the outer membrane. The peptidoglycan layer and lipid bilayer of the cell membrane in gram-positive bacteria determines the electrostatic pull between nanomaterials and bacterial cell surface

Importantly, NM-microbe interaction occurs at the interface between microbial cell surface and NMs, particularly when surface characteristics of each promote such interaction. Electrostatic interaction is the key prominent force facilitating NM binding to bacterial cell surfaces (Fig. 3) [79]. Evidenced by its antimicrobial result, this surface electrostatic interaction changes in accordance with NM surface charge. For example, strong electrostatic forces facilitate binding of positively charged polyethyleneimine coated silver NPs to the negatively charged bacterial outer membrane or cell wall, while electrostatic repulsion is implicated in the diminished microbial killing ability observed with uncoated, citrate or polyvinylpyrrolidone coated silver NPs [80, 81]. Applications of aerosolized silver NPs for respiratory disease remains debatable as current literature is limited and conflicting [82–84]. In more direct electrostatic interaction studies, the amounts of gold (Au) NPs bound to the bacterial cell was quantified [79]. Negligible amounts of AuNPs coated with negatively charged 3-mercaptopropionic acid were detected on bacterial cell surfaces, in contrast to positively charged AuNPs coated with either 3-mercaptopropylamine (MPNH_2_) or poly allylamine hydrochloride (PH), both highly surface bound. Significantly more PH-AuNPs were bound to bacterial cell walls compared to MPNH_2_-AuNPs suggestive that the greater the positive NP surface charge, the stronger the resulting electrostatic interaction [79]. Critically, however, electrostatic interaction is not solely governed by NP surface charge as similar NPs are often reported to elicit differing antimicrobial effects on Gram-positive and Gram-negative bacteria indicating the influence of bacterial cell wall or outer membrane composition on the interaction [73, 79, 85–89]. For example, PH-Au NPs associate sooner with the peptidoglycan layer of gram-positive *Bacillus* versus the equivalent *Shewanella* gram-negative model, protected by the lipopolysaccharide (LPS) layer of its outer membrane [79]. LPS composition varies between gram-negative bacteria, such that smooth LPS microbes have superior AuNP binding that further influences the electrostatic interaction [73, 90]. Through simulation experimentation, PH-Au NPs formed quicker and stronger associations with smooth LPS of *P. aeruginosa* compared to *Escherichia coli* that bears short, rough LPS [90]. The existing variability in the LPS bilayer is therefore likely to be important in defining interaction with NMs. The Gram-positive cell wall also expresses highly diverse cell surface properties that influence NM interaction. Such variations include the presence of surface-layer structures; monomolecular protein arrays that surround the entire outer surface of *Clostridia* and *Bacilli*, as well as the unusual outer membranes of the Mycobacteria (including *Mycobacterium tuberculosis* - TB) that exhibit further variation. This variation includes the expression of multilayered peptidoglycan, covalently linked arabinogalactan and long-chain mycolic acids in their cell membrane that accommodate diverse interaction profiles with a range of NMs [91, 92]. Variation in cell surface composition, penetrative potential or fluidity can occur among bacteria, even within strains of the same species or, indeed, among the same strains under difference environmental conditions or selective pressures with the potential for dynamically changing interactions with NMs [93–95]. NMs may also interact with viral and fungal pathogens causing changes to cell ultrastructure and altering virulence in the latter [95]. In the context of the highly diverse microbial communities, as seen in the microbiome, that further varies in terms of cell surface moieties such as LPS, documented observations clearly indicate that certain microbes are more inclined to bind and interact with NMs compared to others and that each environment (and its microbial make up) should be independently evaluated. [93].

While electrostatic interaction is the most basic interaction between NMs and microbes, NMs also bind bacterial cell surfaces through hydrogen bonds (Fig. 3) [96–98]. Increased hydrogen bond formation is detected in LPS-adsorbed metal oxide NPs including titanium (TiO_2_), aluminium (Al_2_O_3_), and silicon (SiO_2_) oxides [97, 98]. Similarly, infrared spectroscopy corroborates that LPS treated Al_2_O_3_, SiO_2_ and zinc oxide (ZnO) NPs possess detectable hydrogen bonds facilitating interaction with the O-antigen LPS component [96]. Other forces playing key roles in facilitating NM-microbe interaction include *van der Waals* forces and hydrophobic interactions [99]. Graphene nanosheets form strong hydrophobic relationships with the phospholipid tail of Gram-negative outer membranes. This interaction is strong enough to overcome the lipid-lipid pull within bacterial cell membranes resulting in the ability of graphene nanosheets to pierce the membrane and release phospholipids [99–101]. Hydrophobic interaction is also recognised as the major force driving graphene oxide adhesion to environmentally relevant bacteria including *Pseudomonas fluorescens* [102]*.* NMs employed in most research studies are uniformly synthesised and exhibit defined properties such as charge and hydrophobicity. While such NMs are useful to elucidate mechanisms underlying the NM-microbe interaction process, emitted and ambient airborne NMs vary in terms of their physicochemical makeup such as size, charge and chemical composition making direct translation of existing research challenging [103–105]. Additionally, emitted NMs undergo transformation after exposure to common air pollutants such as polycyclic aromatic hydrocarbons (PAH) which in turn further influences interactions with airborne microbes [106]. Given the multi-factorial nature of emitted NMs and NPs, and the biodiversity of the air microbiome, a complex model of NM-microbe interaction is highly challenging to recapitulate in vitro. Specific and directed assessments are therefore critical and should include evaluation of the pre-, pro- and anti-biotic effects of NMs and NPs on specific microbes. This will help uncover the long-term implications and potential beneficial NM or NP-microbe interactions and consequences ranging from maintaining health to causing disease.

#### Deposition of inhaled nanomaterials into the respiratory tract and potential interaction with resident microbiomes

NPs can enter the human body through the airways, skin or by ingestion [107]. A positive correlation between an increased atmospheric particulate concentration and short-term morbidity and mortality has been described epidemiologically [108, 109]. Entry of airborne particles through the respiratory system permits translocation of NPs to other primary organs in small quantities [110–113]. Depending upon the physicochemical characteristics of the inhaled NPs, the respiratory system can be one of the primary sites of toxicity. Most available data focuses on the respiratory effects of ultrafine particle inhalation including dust and black carbon with limited reports on NP induced lung injury [114–120]. Upon inhalation, NPs must overcome physical lung barriers that function to protect against particle deposition, these include mucus production and mucociliary clearance [121]. If NPs persist, they may translocate across the air-blood barrier permitting systemic access to primary organs including the liver, spleen and heart [116–118, 122–128]. However, this is a minor mechanism of clearance for NPs whereas the majority of inhaled NPs are cleared by effective and healthy mucociliary clearance [127, 128]. The key hypothesized mechanism governing NP translocation is endocytosis through the alveolar epithelium [129]. Inhaled NPs also gain systemic access to the central nervous system through the olfactory bulb, bypassing the lungs, with potential neurotoxic consequence [130, 131]. The relevance of this uptake route in humans is expected to be lower than that observed in animal studies because the olfactory mucosa only represents 5% of the total nasal mucosa in humans as opposed to 50% in rats [4].

The lung consists of two functional systems: the conducting airways that include the trachea, bronchi, and bronchioles and the respiratory zone that consist of the alveoli and all structures involved in gas exchange [132]. The human lung contains 500 million alveoli with an estimated surface area of 140 m2 [133, 134]. The pseudostratified epithelium constituting the barrier to bloodstream absorption differs between the conducting airways and alveoli. The airways have a gradually thinning columnar epithelium, ranging from 5 mm to 1 mm, protected by overlying mucus and mucociliary clearance [132, 135–137]. In contrast, the alveoli consist of thin, single cell monolayers with less than a 400 nm distance between air in the alveolar lumen and capillary blood flow. The large alveolar surface area and tight air–blood barrier makes alveoli more susceptible to the effects of inhaled NPs when compared to the airways [136]. The site, extent and efficacy of particle deposition after inhalation is influenced by several factors including (a) particle size, density, surface properties or shape; (b) the anatomy of the airways and alveolar structure; and (c) ventilation such as breathing pattern (including breath-holding and the presence of expiratory flow limitation), flow rates and tidal volume all of which dictate deposition pattern, airflow velocity and NP resident time in the respiratory tract [138–141].

**Fig. 4:**
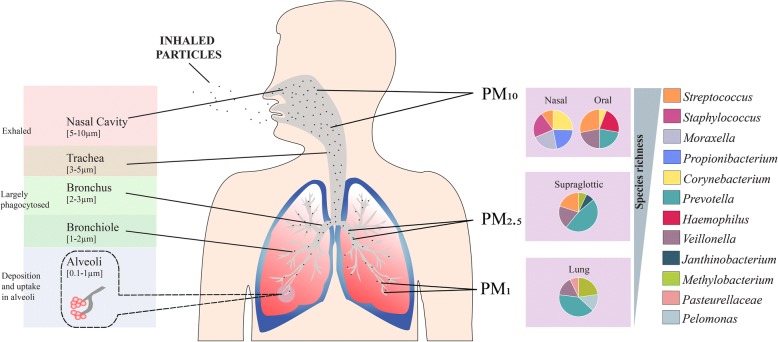
Size dependent regional deposition of inhaled nanoparticles and their interaction with resident lung microbiome. The diameter of the airway is indicated, as are the deposition sites of various inhaled nanoparticles of differing size (PM_10_ PM_2.5_ and PM_1_) which locate to specific regions of the respiratory tract. Regional variation in the microbiome is also illustrated across different regions of the airway. A proportional representation of the top five abundant bacterial taxa at each site is indicated. Decreasing species richness is observed in the lower airway

Particle size or aerodynamic diameter is a variable that depends on composition and fabrication technique. Successful deep lung deposition requires particles to be small enough to avoid inertial impaction in the upper airways which permits their passage into lower airways, but large enough to avoid being exhaled [142, 143]. Important definitions by the World Health Organization (WHO) make a clear distinction between inhalable, thoracic and respirable dust [144]. Inhalable dust is that which can reach upper airways including the mouth, nose and throat (PM_10_ < 10 μm). The thoracic fraction is smaller and can penetrate bronchi and upper bronchioles (PM_2.5_ < 2.5 μm). Respirable dust contains the smallest particles (PM_1_ < 1 μm) able to enter alveoli and access the systemic circulation (Fig. 4) [145–148]. Optimal particle size to achieve delivery into alveoli is 1–3 μm [149]. Airborne particles of < 1 μm are exhaled in most cases because of low inertia, however particles < 500 nm have greater overall lung deposition because of their increasing diffusional mobility while smaller ultrafine particles (< 100 nm) appear to settle effectively in the alveolar region with a fractional deposition of ~ 50% [136, 149–152]. These observations are confirmed by clinical studies evaluating laboratory-generated ultrafine particles [125, 153–155]. High-deposition efficiency in healthy subjects is observed and gets worse in those with chronic inflammatory respiratory disease such as asthma and COPD – a condition noted for increased microbial load in the lung [156–162]. NP deposition within the respiratory tract is further dictated by diffusional displacement of the thermal motion of air molecules interacting with particles in both inhaled and exhaled air streams [163, 164]. Nanofibers with small diameters therefore will penetrate deeper while longer fibres (> 20 μm) will predominantly locate to the upper airways [165, 166]. The respiratory microbiome exhibits topographical variation from the nasal and oral cavity through to the supraglotic space and the lower lung regions (Fig. 4) [41]. This bears relevance to the deposition of nanoparticles, which may reach different regions of the respiratory tract and thus encounter variable microbiota with which they can potentially interact (Fig. 4). The variation seen in the microbiome of the respiratory tract accords with the ‘adapted island model’ of lung biogeography with decreasing diversity seen as one descends the respiratory tract [40]. In infectious disease states such as Tuberculosis (TB) - a highly contagious infection and devastating chronic respiratory disease caused by *M. tuberculosis* - immunological events in the airways and the host microbiota have been demonstrated to influence infectivity [167]. The dysbiosis of airway microbiota in pulmonary tuberculosis plays a key role in its pathogenesis, complicating the intimate immuno-physiological interaction between pathogen and host [167–169]. The distribution of TB in term of abundance and location in the airway may influence how NPs deposit and interact, which would be of particular importance given the global burden of TB, and its high prevalence in developing countries where nanomaterials are being introduced on industrial scales [170, 171]. The nasal and oral cavities of healthy individuals harbour abundant bacterial genera such as *Corynebacterium* and *Stapylococcus* representing core microbiota of these anatomical sites while higher densities of distinct taxa including *Prevotella* and *Methylobacterium* are noted in the lower airways [41, 172] (Fig. 4). As such, the size of a given nanomaterial will determine its site of deposition within the respiratory tract and, consequently, the range of microbial taxa with which it can potentially interact with in vivo*.*

#### Immunological consequences of inhaled nanomaterials and their interaction with the respiratory microbiome

**Fig. 5:**
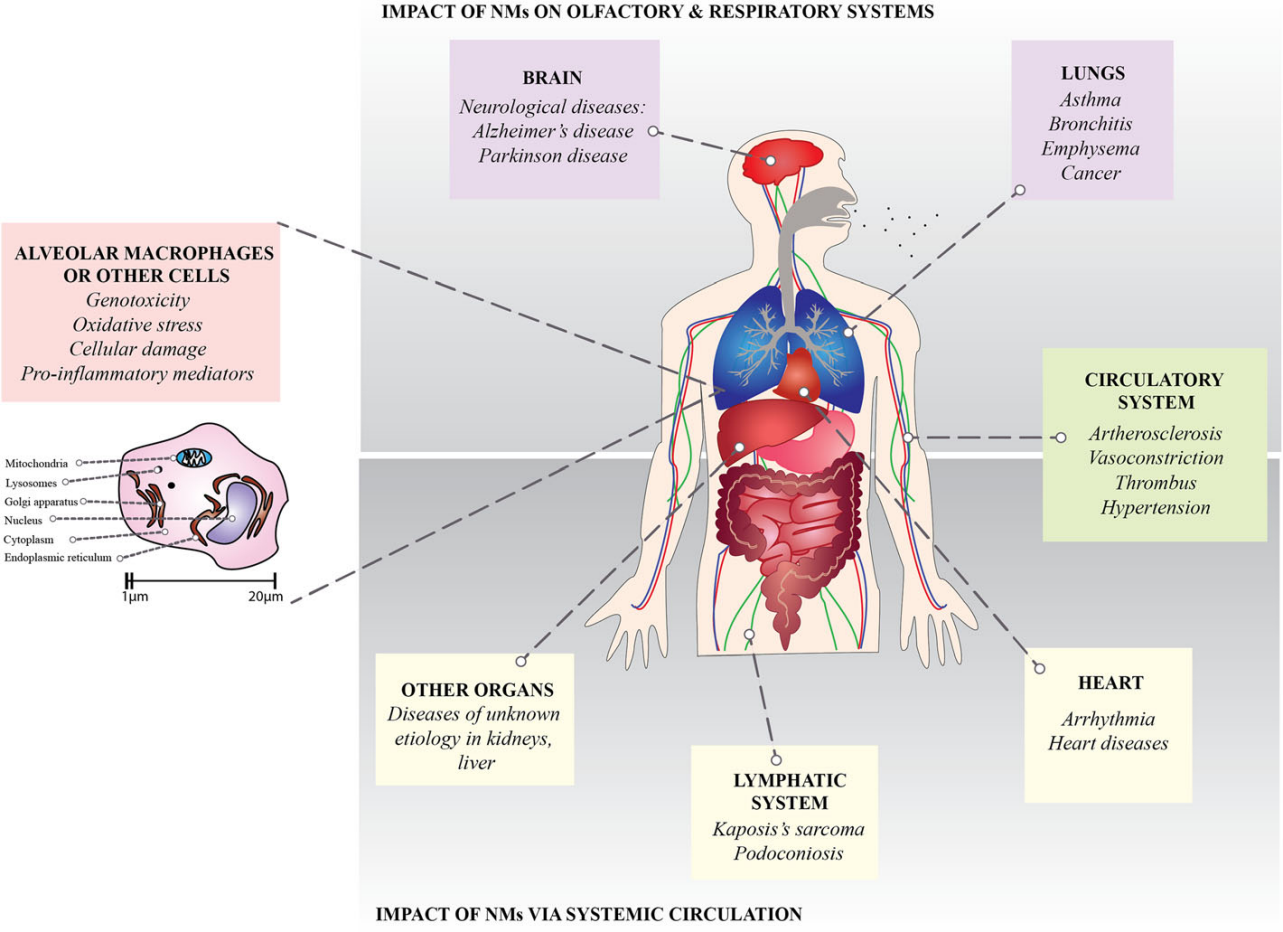
A summary of the health consequences from inhaled nanomaterials (NMs). Following inhalation, NMs enter airway cells, lymph nodes and the circulatory system including an ability to cross the blood-brain barrier through the olfactory system. They can accumulate in organs including the lungs, liver and brain leading to a range of disease states. Some NMs influence the human immune system and interfere with phagocyte (e.g. macrophage) function

NPs induce systemic effects resulting in cardiorespiratory morbidity and mortality (Fig. 5) [117, 120, 157–159, 161, 173–178]. While this is widely accepted, mechanisms driving these effects remain unclear. Considerable concern persists over the ability of NPs to cross the alveolar air-blood barrier which allows systemic access and the potential for adversely impacting a wide range of cells and organ systems (Fig. 5).

Upon deposition of inhaled NMs within lung-lining fluid, separate bio-kinetics dictate either their lung absorption or clearance [179]. Fluid-soluble NMs undergo chemical dissolution in situ whilst low molecular weight hydrophobic and insoluble NMs are absorbed through the lung epithelium by passive diffusion [113, 180]. Diffusivity through alveoli is faster than the small airways and solutes are cleared systemically into the bloodstream and lymphatic circulation. Through contact with the epithelial fluid lining (ELF), NMs come into close proximity of host defence mechanisms and immune cells, each of which play a role in NP–cellular interaction [164]. Innate immune mechanisms such as the mucociliary escalator is the first line of defence removing deposited NPs to maintain relative lung mucosal “sterility” [181, 182]. NPs consisting of slowly dissolving or insoluble NMs will be swept away by cilia toward the larynx, where they are either swallowed or expelled by coughing. The clearance of insoluble NPs from alveoli is mediated by macrophage phagocytosis and endocytosis, a process under routine surveillance by 12–14 alveolar macrophages in each of the 500 million alveoli [133, 183]. While particles < 0.26 μm may escape phagocytosis, they interact with the epithelium, and the endocytic events that follow are regulated by clathrin-coated pits, caveolae and scavenger receptors (SR) including SR-A. Caveolae and coated pits preferentially transport small and large particles respectively, but this assertion lacks in vivo validation [184]. Transport through pores similar to that occurring in lung–blood nutrient exchange is another key dissemination route for inhaled NMs. The inspiratory expansion and expiratory contraction of lung alveoli leads to the opening and closing of the caveolae. These openings measure between 40 and 100 nm and can transport macromolecules including NMs across the alveolar-capillary barrier [185]. A reactive NM surface can further propagate chemical interactions between NPs and cellular plasma membranes by inducing lipid peroxidation at its interface. This causes change to membrane permeability further compromising the host immune response [186]. NPs < 34 nm with a negative surface charge (including pollutants and carcinogens) possess a demonstrable ability to translocate into the systemic circulation through mediastinal lymph drainage and are therefore retained as compared to smaller (< 6 nm) zwitterionic NPs that may be cleared by the kidneys [124]. NP-driven mechanisms inducing a systemic immune response are largely unresolved but have been hypothesized as a secondary rather than a primary effect of NP exposure. Inhaled NPs however are known to directly induce airway inflammation by promoting release of pro-inflammatory cytokines and chemokines from the lung epithelium and accompanying immune cells including those recruited systemically [187–199]. Precise mechanisms for this NP-induced pro-inflammatory airway response remains unclear but a proposed suggestion is an immunogenic surface NP antigen with similarity to endogenous biomolecules [200]. Depending on size and surface reactivity, NPs are therefore dispersed, deposited and then possibly absorbed, all processes inducing a host immune airway and systemic response.

Inhaled NPs interact with the respiratory microbiome further inducing a damaging immune response. Existing work demonstrates correlation between the amounts of bound NPs to bacteria and the observed bactericidal activity [73, 77, 78, 90]. Bound NPs can rupture bacterial cells, lead to alteration in membrane potential, release ions at the bacterial surface and potentially generate cellular membrane reactive oxygen species (ROS). LPS, a major component of the outer membrane of gram-negative bacteria is important in facilitating NP binding to bacterial surfaces and inducing inflammatory responses in cells expressing toll-like receptor 4 (TLR4) [73, 77, 78, 90, 201]. TLR4 is expressed on many different cell types but monocytes and macrophages are most reactive and abundant in the airway. In response to LPS, classical activation (M1 macrophages) are cytocidal producing a range of inflammatory mediators including interleukin (IL)-1β, IL-6, IL-8, and tumour necrosis factor (TNF)-α [201, 202].

LPS, being ubiquitous in nature, is a common contaminant in chemicals and glassware during NP preparation because of its heat stable properties and intrinsic resistance to sterilisation [203–208]. Non-inflammatory NPs may acquire inflammation-inducing capacity by simply adsorbing LPS onto their surface. LPS-coated NPs are new entities, with a functional profile differing from LPS or NPs alone. LPS-coated NPs appear to maintain capacity to induce inflammatory responses including NFκB activation, production of IL-1β and IL-8 but lose their concomitant anti-inflammatory effect (expression of IL-1Ra). LPS NP surface adsorption therefore affects the pro-inflammatory properties of both LPS and NPs [209]. This immune-inflammatory-microbial relationship is relevant to pulmonary *P. aeruginosa* and other gram-negative pathogens expressing LPS highlighting the importance of understanding NP-microbe interactions at source owing to their influence on the host-microbe-environmental interplay in individuals exposed to inhaled NMs.

#### Toxicological consequences of inhaled nanomaterials and their interaction with the respiratory microbiome

As we learn more about the human microbiome, a focus shift toward the study of its toxicological effect in relation to NMs exposure has evolved in nanotoxicology focused research. NMs including nanosilver and nanotitanium dioxide, both antibacterial agents, have been introduced successfully into commercial products, however, unintended consequences of their use have been recognised including the emergence of antimicrobial resistance. Most work in this field has focused on the gut microbiome, largely owing to its manipulation by antibacterial agents, where the oral route of administration is routinely used [210, 211]. In other related work, skin microbiota has been examined, particularly in the context of NM use as antibacterial agents in wound dressings, however, dedicated research focused on the respiratory microbiome is lacking [212]. Many studies have described the effects of NMs on individual microbes rather than the collective ‘microbiome’ community, and most available research has been restricted to animal models, making human translation challenging largely because of using a setup that forgoes the complexity of the in vivo state [213]. While animal models provide the next best alternative to human studies, the field has lacked work devoted to NM-microbiome relationships. In fact, preliminary work on the influence of carbon nanotubes, silica and liposomes on the gut microbiome in animals is reported but limited in their scope [210, 211]. The best studied NP in relation to the microbiome remains nanosilver, shown in gut microbiome studies to confer dose-dependent dysbiosis [214]. Importantly, however, existing work remains conflicting with several studies illustrating inconsistent changes to *Firmicutes, Lactobacillus* and *Bacteroidetes* [215]. Other work further demonstrate increases in gram-negative bacteria including some potentially pathogenic taxa [216]. Despite the inconsistency, interestingly, nanosilver stabilised with polyvinylpyrrolidone did not cause any toxic effects in four-week old rats exposed to a variety of dosing over 28 days [217]. Similarly, a 28-day exposure in pig models showed no effect on microbiome diversity or relative species abundance in the gut microbiome regardless of NM size or and coating [218]. Study variable results may be explained by the different animal models employed, contrasting methodologies used to assess the microbiome or the simple fact that there may be little or no effect of the NPs themselves. Critically, however, an area for further work remains the physicochemical properties of the NMs themselves, their dose and duration of exposure. Critically, there are also likely differences between the human impact of NPs and currently employed animal models however future work may potentially reduce such heterogeneity by assessing NP-microbiome-immune interactions using in-vitro model systems such as 3D cell culture, primary cell cultures or fully differentiated air-liquid interface approaches, all areas lacking evidence in the current literature.

NP as substitutes for antibiotics in the animal farming industry has been explored and provides further insight into relationships with host microbiomes. Two separate investigations in pigs showed that silver and copper-loaded chitosan NPs increased animal body weight hypothesized to be a result of the NPs antibacterial properties which decreased gut microbial loads [219, 220]. While this may be of benefit in the farming industry, human translation would equate to obesity and importantly the widespread use of these agents to promote weight gain in agricultural settings with poorly defined mechanisms and potential impact on the microbiome raises concern over potential health consequences in humans who consume these foods.

Human studies in this field, like most nanotoxicology focused research, has lagged behind that conducted in the more accessible animal model systems. Despite this, indirect assessments on humans have been performed, for instance measuring gas release from human stool obtained from subjects after 48 h of nanosilver ingestion provides a limited surrogate for gut microbiome activity [221]. The observed effects were quantitatively comparable but qualitatively different when compared to silver chloride ingestion. Nanosilver exhibits a significant antibacterial effect resulting in a 22% reduction of gas produced at the highest tested concentrations. In addition, it modifies fatty acid methyl ester profiles even at modest levels. Correlating with some mice studies, abundance of *Bacteroides ovatus* was significantly reduced while the gram-negative species, *Raoultella* and *E.coli* were increased [221].

While gut microbiome studies remain sparse in human systems, the skin microbiome has been better studied largely as a consequence of NP use as antibacterial agents in wounds and personal care products. A recent review covers potential strategies targeting the skin microbiome using NMs [212]. Use of nanosilver as a regulator of skin microbiome composition is an established strategy to combat acne and has been shown to be inhibitory against *Staphylococcus aureus* (*S. aureus*), *P. aeruginosa*, *Streptococcus pyogenes* and *E.coli* [222, 223]. ZnO NPs, agents targeting skin infection, illustrates anti-biofilm abilities with strong demonstrable effects against *S.aureus* [224]. Intradermal administration of ZnO NPs significantly reduced the occurrence, bacterial load and inflammation associated with skin infection in a mouse model while improving skin architecture [224]. It is important however that such effects, mediated by Zn^2+^ alone, occurred because of wound dissolution of ZnO NPs.

The NM and NP associated effects on the lung microbiome are less well characterised and little is known outside preliminary animal focused work [18, 225]. Research focused on the gut demonstrates selective shifts in microbial community structure and function following the ingestion of environmental pollutants. For example, arsenic-treated mice experience reductions in gut *Firmicutes* but not *Bacteroidetes* [226]. Bacteria, importantly, have been known for decades to contribute to the biotransformation of environmental metals such as arsenic [227]. Non-estrogenic by-products of combustion pollutants such as PAHs may also be converted to compounds with estrogenic-like activity by bacteria from the human colon [228]. Similar reactions potentially occur with the lung microbiome where toxicity of inhaled pollutants like metals and PAHs, is influenced by microbiome-mediated chemical conversion. This is further evidenced by administration of antibiotics to mice, where an altered airway response results after ozone gas exposure [229].

Lung microbiome studies have largely focused on its exposure to antimicrobial NPs and their associated community dysbiosis, with only limited reports on non-antimicrobial related NMs and NPs [225]. Future work is required to understand the effects of chronic exposure of non-antimicrobial NMs and NPs on the airway microbiome given the ability of such particles to penetrate the lower respiratory tract. Understanding the interaction between NMs and the airway microbiome is a fledgling but growing field necessitating high quality research including well-designed experiments and complementary epidemiological studies to provide a clearer and more comprehensive understanding of clinical, immunological and toxicological consequence [18].

## Conclusion

The human microbiome is a complex and diverse ecosystem with key symbiotic functions across a range of human organ systems. Recently, the interaction between NMs and the human microbiome is recognized as a key determinant for human health and disease particularly of the respiratory system. While some existing work focused on gut and skin microbiomes, much of which is restricted to animal models, our understanding of similar relationships in the airway requires attention owing to the vast range and complexity of inhaled NMs that humans are now exposed to. Future studies in this evolving field must be well designed and account for the model system used, with preference for in vivo human studies but also consider key endpoints, measurements and NP dosing. Lessons from past work include differential responses and relationships even from genetically homogenous animal models which upon translation to the human setting add significant complexity to study design. Human genetic diversity influences microbiome communities and their structure as do environmental and lifestyle factors including air quality and diet. Performing meaningful and translational studies in this field will likely have impact for public health policies with regulatory consequence. The influx of ‘big data’ from high throughput “-omics” approaches will likely further increase our understanding of microbiome function in the context of NM exposure – a relationship with clinical relevance that requires a holistic approach, including immunology, toxicology and environmental approaches.

## Abbreviations

Al_2_O_3_: Aluminium
Au: Gold
DNA: Deoxyribonucleic acid
*E.coli*: *Escherichia coli*
ELF: Epithelial fluid lining
IL: Interleukin
LPS: Lipopolysaccharide
m^2^: Metre squared
MPHN_2_: 3-mercaptopropylamine
NFκB: Nuclear factor kappa-light-chain-enhancer of activated B cells
nm: Nanometre
NMs: Nanomaterial
NPs: Nanoparticles
PH: Poly allylamine hydrochloride
PM_0.1_: Particulate matter 0.1 μm
PM_1_: Particulate matter 1 μm
PM_10_: Particulate Matter 10 μm
*P. aeruginosa*: *Pseudomonas aeruginosa*
PM_2.5_: Particulate matter 2.5 μm
ROS: Reactive oxygen species
*S. aureus*: *Staphylococcus aureus*
SiO_2_: Silicon oxide
SR: Scavenger receptors
TB: Tuberculosis
TiO_2_: Titanium
TLR-4: Toll-like receptor 4
TNF-α: Tumour necrosis factor alpha
ZnO: Zinc oxide
μm: Micrometres
